# Treating EGFR‐mutant nonsmall cell lung cancer is no longer a one‐size‐fits‐all approach

**DOI:** 10.3322/caac.70027

**Published:** 2025-08-04

**Authors:** Lauren J. Antrim, Jyoti Malhotra

**Affiliations:** ^1^ City of Hope Comprehensive Cancer Center Duarte California USA

Just over 2 decades ago, Lynch et al. published a landmark article in which they elucidated that activating mutations in the epidermal growth factor receptor (*EGFR*) positively correlated with clinical responses to the first‐generation tyrosine kinase inhibitor (TKI), gefitinib, in patients with nonsmall cell lung cancer (NSCLC).^1^ Although estimates vary, it is believed that up to one third of cases of NSCLC may harbor these mutations.^2^ Over the last 20 years, great strides have been made to improve both the efficacy and the tolerability of therapies targeting EGFR, with fourth‐generation EGFR TKIs in development. In this edition of *CA: A Cancer Journal for Clinicians*, Borgeaud et al. provide an exceptionally detailed review of *EGFR*‐mutant NSCLC that describes the molecular underpinnings of this oncogenic driver, discusses modern treatment approaches, and explores the gamut of clinical situations that can be encountered while treating a patient with *EGFR*‐mutant NSCLC.^3^

Although Borgeaud and colleagues explicitly do not cover the management of *EGFR* exon 20 insertions, they do provide some brief insights into P‐loop and αC‐helix compressing (PACC or *uncommon*) mutations and present a comprehensive review that can be a useful tool for clinicians treating patients with *common* *EGFR* mutations (L858R, exon 19 deletions) across all stages of presentation. Studies investigating the effectiveness of immunotherapy and vascular endothelial growth factor (VEGF) inhibitors to early phase trials targeting potential acquired mutations on EGFR TKIs are detailed. For clinicians treating patients with early stage or locally advanced NSCLC, their article analyzes pivotal studies, such as ADAURA and LAURA (ClinicalTrials.gov identifiers NCT02511106 and NCT03521154, respectively), which led to osimertinib (third‐generation TKI) becoming the standard of care in the adjuvant and postchemoradiation setting, respectively.^4^, ^5^ Furthermore, crucial ongoing studies are highlighted, such as NeoADAURA (ClinicalTrials.gov identifier NCT04351555), which is investigating the incorporation of osimertinib in the neoadjuvant setting.^6^ Potential considerations for less straight‐forward situations, such as metastatic recurrence while on or after adjuvant osimertinib, are also discussed.

In recent years, treatment of *EGFR*‐mutant NSCLC in the metastatic setting has grown increasingly complex, with multiple treatment regimens now approved. Borgeaud et al. provide a detailed overview of available first‐line regimens and important treatment considerations. With the initial publication of the FLAURA trial (ClinicalTrials.gov identifier NCT02296125) in 2017 and subsequent study updates, osimertinib became the clear choice for first‐line systemic therapy in the metastatic setting, with an improvement in median progression‐free survival and median overall survival (OS) compared with first‐generation TKIs and, importantly, a favorable toxicity profile.^7^ Subsequently, in 2023, FLAURA2 (ClinicalTrials.gov identifier NCT04035486) demonstrated an improvement in median progression‐free survival using the combination of osimertinib plus chemotherapy (platinum and pemetrexed) compared with osimertinib monotherapy in the first‐line setting,^8^ as did MARIPOSA (ClinicalTrials.gov identifier NCT04487080) in 2024 using the combination of amivantamab (an EGFR and MET bispecific antibody) plus lazertinib (third‐generation EGFR TKI).^9^ While long‐term follow‐up and survival data are maturing in both combination regimens, there has been a trend toward an OS benefit with the FLAURA2 regimen^10^ and an improvement in median OS with the MARIPOSA regimen (predicted to exceed 1 year over the osimertinib monotherapy regimen).^11^ With a potential OS benefit, will combination therapy be the new go‐to front‐line therapy?

Identifying which patients may benefit most from combination therapy is of great importance. There are various disease‐related factors that may be considered *high‐risk* for earlier progression on osimertinib monotherapy, such as the presence of brain metastasis or the L858R mutation, which Borgeaud et al. describe at length and provide an excellent visual representation (see Figure 5). However, a patient is not an amalgamation of their disease, and the potential impact of toxicity of proposed regimens should be taken in to account as part of shared decision making.

Although osimertinib has less toxicity than EGFR TKIs from earlier generations, grade 3 or greater adverse events were still reported in 27%–43% of patients on osimertinib monotherapy in FLAURA, FLAURA2, and MARIPOSA.^7^, ^8^, ^9^ On the osimertinib monotherapy arm in those studies, 26%–40% of study participants did not go on to receive subsequent therapy.^7^, ^8^, ^9^ However, patients on clinical trials may not reflect the real‐world patient population in which patients with lung cancer may have higher incidence of comorbidities and worse performance status. One single‐institution, real‐world study reported that over one half of patients who received front‐line osimertinib would not have met eligibility for inclusion in the FLAURA trial.^12^ Another real‐world, retrospective study in the United States demonstrated that only 39% of patients with metastatic NSCLC on first‐line osimertinib went on to receive a second line of therapy.^13^ Therefore, it is crucial to make efforts to ensure the success of whatever regimen is used because it very well may be the only systemic therapy many patients ever receive.

One treatment‐agnostic intervention that is known to support patients with metastatic NSCLC is early integration of the palliative care team, which improves quality of life and symptom control for many patients. Moreover, the median OS is longer with the incorporation of early palliative care despite reduced aggressive end‐of‐life care in these patients.^14^ Because both combination regimens were found to have increased rates of grade 3 or greater adverse events than their comparator arm (64% in FLAURA2 and 75% in MARIPOSA), additional considerations should be made to manage toxicity.^8^, ^9^ Supportive care to mitigate side effects from chemotherapy should be administered with the FLAURA2 regimen as clinically indicated, such as antinausea medications and granulocyte‐colony–stimulating factor for neutropenia prophylaxis in patients with high risk. With the MARIPOSA regimen, significant efforts have been made to make the regimen more tolerable, with studies such as SKIPPirr (ClinicalTrials.gov identifier NCT05663866), which used steroids to minimize infusion reactions during initial infusion administration, as well as COCOON (ClinicalTrials.gov identifier NCT06120140), which combined oral antibiotics and topical agents to prevent dermatologic toxicities.^15^, ^16^ These supplemental therapies present their own challenges, such as polypharmacy, but should be taken in to account in the care of in any patient initiating treatment on amivantamab and lazertinib.

In addition to side effects, additional psychosocial stressors are important aspects of care as well. In choice of therapy, time toxicity varies greatly between the first‐line regimens. The amount of time a patient has to interact with the health care system on an oral TKI is significantly less than with regular intravenous infusion treatments on the FLAURA2 regimen and, to an even a greater extent, with the MARIPOSA regimen. These long and frequent visits potentially affect time off work for patients and caregivers and may contribute to health care fatigue. Furthermore, financial toxicity is an important aspect of treatment. One recent study evaluated the comprehensive cost per patient for the first year of treatment with the MARIPOSA and FLAURA2 regimens, including the costs of treatment acquisition, administration, disease management, and adverse event management in patients with locally advanced or metastatic, *EGFR*‐mutant NSCLC treated in the United States. The MARIPOSA regimen was identified as roughly three times the cost of the FLAURA2 regimen with both Medicare ($220,129 vs. $79,348) and private insurance ($662,200 vs. $235,709).^17^

In conclusion, with the evolving landscape of EGFR‐directed therapies, there is no longer a clear first‐line choice in the metastatic setting for *EGFR*‐mutant NSCLC. A clinician must weigh efficacy with toxicity and actively support patients throughout their treatment journey. The outstanding review from Borgeaud and colleagues provides a high‐yield reference for these treatment considerations as well as a plethora of other clinical scenarios that clinicians may face while treating patients with *EGFR*‐mutant NSCLC.

## CONFLICT OF INTEREST STATEMENT

Jyoti Malhotra reports personal/consulting fees from AstraZeneca and Johnson & Johnson outside the submitted work. Lauren J. Antrim disclosed no conflicts of interest.
